# Modifications of cell wall polymers in Gram-positive bacteria by multi-component transmembrane glycosylation systems^☆^

**DOI:** 10.1016/j.mib.2021.01.007

**Published:** 2021-04

**Authors:** Jeanine Rismondo, Annika Gillis, Angelika Gründling

**Affiliations:** 1 Department of General Microbiology, GZMB, Georg-August-Universität Göttingen, 37077 Göttingen, Germany; 2 Section of Molecular Microbiology and Medical Research Council Centre for Molecular Bacteriology and Infection, Imperial College London, London SW7 2AZ, United Kingdom

## Abstract

Secondary cell wall polymers fulfil diverse and important functions within the cell wall of Gram-positive bacteria. Here, we will provide a brief overview of the principles of teichoic acid and complex secondary cell wall polysaccharide biosynthesis pathways in Firmicutes and summarize the recently revised mechanism for the decoration of teichoic acids with d-alanines. Many cell wall polymers are decorated with glycosyl groups, either intracellularly or extracellularly. The main focus of this review will be on the extracellular glycosylation mechanism and recent advances that have been made in the identification of enzymes involved in this process. Based on the proteins involved, we propose to rename the system to multi-component transmembrane glycosylation system in place of three-component glycosylation system.

## Highlights

• Cell walls of Gram-positive bacteria have evolved to produce different glycopolymers.
• LTA is glycosylated by a multi-component transmembrane glycosylation system.
• WTA and complex cell wall polysaccharides can be glycosylated intracellularly and extracellularly.
• GT-C-fold glycosyltransferases can be predicted by structural homology analysis.

## Graphical abstract

**Figure:**
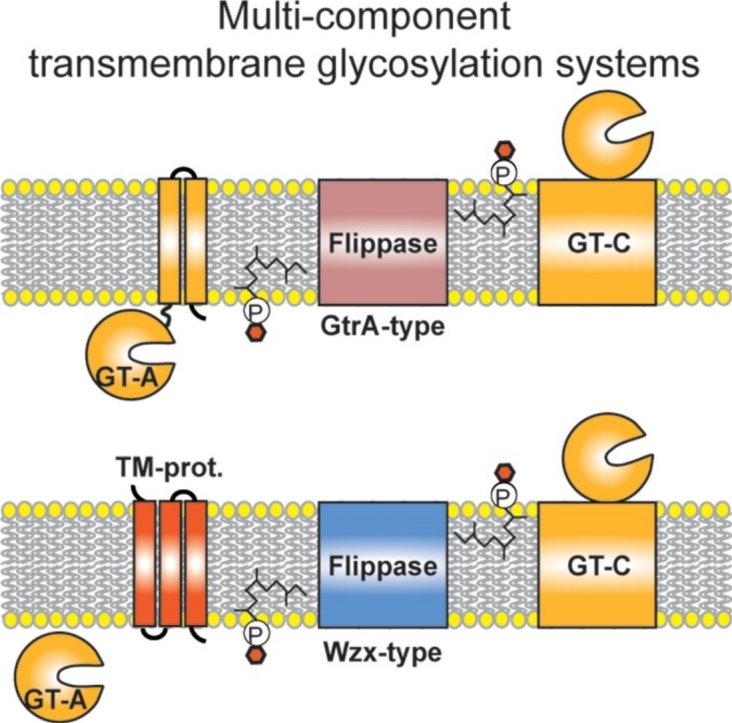

**Current Opinion in Microbiology** 2021, **60**:24–33

This review comes from a themed issue on **Special section on bacterial cell wall synthesis**

Edited by **Jean-François Collet** and **Angelika Gründling**

For complete overview of the section, please refer to the article collection, “Special section on Bacterial cell wall synthesis”

Available online 9th February 2021

**https://doi.org/10.1016/j.mib.2021.01.007**

1369-5274/© 2021 The Authors. Published by Elsevier Ltd. This is an open access article under the CC BY license (http://creativecommons.org/licenses/by/4.0/).

## Introduction

Gram-positive bacteria belonging to the phylum Firmicutes are of industrial and medical relevance. Active research over many years has led to a detailed understanding of the synthesis and function of their cell wall. Most members of the Firmicutes have a ‘typical’ Gram-positive cell wall, composed of a thick peptidoglycan layer (for recent advances in its synthesis see review by Ducret and Grangeasse in this issue) and secondary cell wall polymers, including teichoic acids (TAs) and more complex secondary cell wall polysaccharides (SCWPs). TAs are further grouped into lipoteichoic acids (LTAs), which are anchored to the outside of the bacterial membrane, most often to a glycolipid, and wall teichoic acids (WTAs), which are covalently linked to peptidoglycan. LTA and WTA are usually polymerized by different sets of enzymes and in different locations: LTA on the outside of the membrane and WTA in the cytoplasm. Here, we will briefly describe the canonical TA synthesis models, including an updated model for their decoration with d-alanines and two main synthesis pathways of SCWPs, and then focus on the mechanism and function of the known enzymes required for the extracellular decoration of TAs and SCWPs with sugar residues. Finally, we will highlight what, in our view, are some of the important outstanding questions in the field.

## Synthesis of lipoteichoic acid and a revised mechanism for its modification with d-alanines

**Figure 1:**
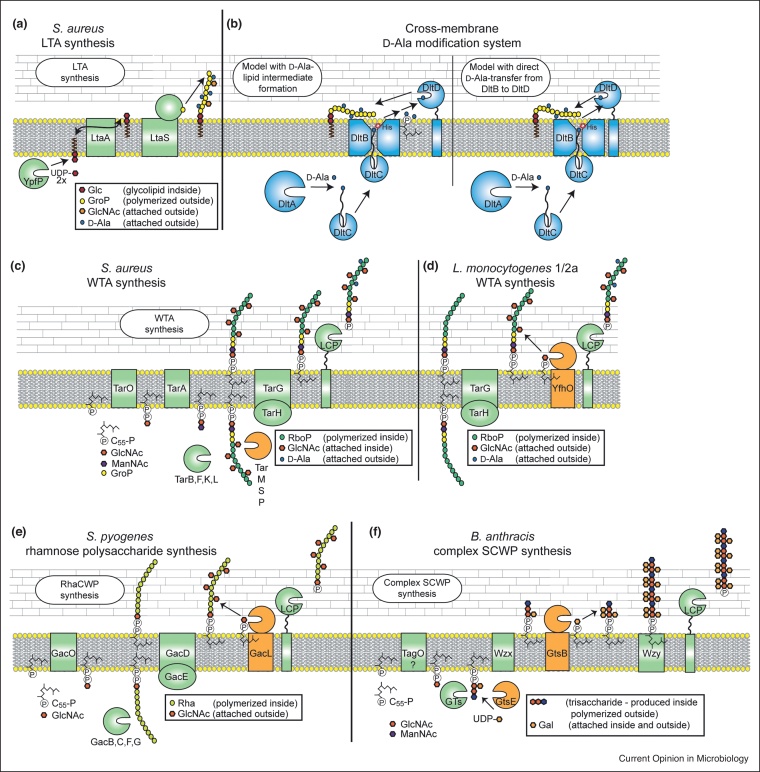
Schematic representation of LTA synthesis, models for the cross-membrane d-alanine modification process and examples of WTA and complex SCWP synthesis pathways. **(a)** Glycolipid anchor and type I poly-glycerolphosphate LTA synthesis in *S. aureus*. The glycolipid Glc_2_-DAG is produced by YpfP using UDP-glucose as substrate and flipped to the outer leaflet of the membrane by LtaA. The GroP polymer is produced by LtaS-type enzymes on the outside of the cell. **(b)** Current models for the cross-membrane d-alanine modification process of LTA. An alanine is attached by DltA to the phosphopantetheine prosthetic group of the carrier protein DltC. DltC binds to DltB, which is thought to transfer the d-alanine onto either a lipid carrier (left side) or directly onto DltD (right side), which then transfers it onto the LTA polymer. **(c)** RboP WTA synthesis in *S. aureus*. The RboP WTA polymer is synthesized in the cytoplasm, modified intracellularly by the glycosyltransferases TarM, TarS and/or TarP, exported and attached to the peptidoglycan layer. **(d)** RboP WTA synthesis in *L. monocytogenes* serotype 1/2a strains. Similar to *S. aureus*, the RboP WTA polymer in *L. monocytogenes* serotype 1/2a strains is polymerized intracellularly, but then thought to be exported and glycosylated extracellularly using a multi-component transmembrane glycosylation system. **(e)** Rhamnose cell wall polysaccharide (RhaCWP) synthesis and glycosylation in *S. pyogenes*. The cell wall polymer is produced in the cytoplasm, exported and glycosylated extracellularly using a multi-component transmembrane glycosylation system. **(f)** Complex SCWP synthesis and glycosylation in *B. anthracis*. The polymer is thought to be produced using a Wzx/Wzy-enzyme translocation and polymerization pathway. Small polymer subunits are produced intracellularly, partially glycosylated intracellularly, transported across the membrane and further glycosylated via a multi-component transmembrane glycosylation system, before polymerization and attachment to the peptidoglycan layer.

The prototype LTA is a poly-glycerolphosphate (GroP) polymer linked to a glycolipid anchor and referred to as type I LTA. Glycolipid anchor synthesis is well-characterized in *Staphylococcus aureus* and takes place within the bacterial cell. The glycosyltransferase YpfP produces the glycolipid diglucosyldiacylglycerol (Glc_2_-DAG) using UDP-glucose as substrate, which is subsequently transported to the outside of the cell by the multimembrane spanning protein LtaA (Figure 1a) [1,2]. The structure of LtaA was recently determined and its lipid flipping activity confirmed *in vitro* [3]. The actual GroP chain is polymerized on the outside of the cell by LtaS-type enzymes using the phospholipid phosphatidylglycerol as a substrate (Figure 1a) and subsequently modified with d-alanine residues [4]. Four proteins, DltA-DltD, are essential for this modification [5^•^]. DltA charges the phosphopantetheine prosthetic group of the carrier protein DltC with an alanine residue. Based on the recently determined structure of the DltB protein, which belongs to the membrane-bound *O*-acyltransferase (MBOAT) family of proteins, and a proposed catalytic function of DltD, two potential and updated mechanisms for the decoration of LTA with d-alanines have been proposed (Figure 1b) [5^•^,6^•^]. In these models, the DltC carrier protein binds on the cytoplasmic side of the membrane to DltB [6^•^]. The d-alanine is then transferred by DltB to a lipid carrier (proposed to be undecaprenylphosphate (C_55_-P) or phosphatidylglycerol) and subsequently by DltD to the LTA polymer on the outside of the cell (Figure 1b, left panel). Alternatively, DltB transfers the d-alanine directly to DltD, which then moves it onto LTA (Figure 1b, right panel) [5^•^]. A Ser-His-Asp triad is thought to form the catalytic site in DltD [5^•^] and a His residue in DltB located in a cleft accessible from the outside of the cell has been suggested to serve as a catalytic residue [6^•^]. Furthermore, DltB is thought to function as ‘channel protein’ allowing the phosphopantetheine-d-Ala group bound to DltC reach partway through the membrane and has also been proposed to function as LTA acceptor binding protein [6^•^].

## Synthesis of wall teichoic acid and complex secondary cell wall polysaccharides

In addition to LTA, Firmicutes bacteria produce WTAs or SCWPs, and, in some cases, both. The best studied WTAs are ribitolphosphate (RboP) or glycerolphosphate (GroP) polymers, whereas well-studied examples of more complex SCWPs include the rhamnose polysaccharide found in *Streptococcus pyogenes* and *Streptococcus mutans*. In contrast to type I LTA, synthesis of WTA and SCWPs is initiated in the cytosol. In the case of WTA, a linker unit is produced by TarO (also referred to as TagO in some bacteria) and TarA (or referred to as TagA), which add phospho-*N*-acetylglucosamine (phospho-GlcNAc) and *N*-acetylmannosamine (ManNAc) onto the lipid carrier C_55_-P to generate C_55_-PP-GlcNAc-ManNAc (Figure 1c). Next, the GroP and RboP backbone is polymerized in the cytosol by a number of Tar (or Tag) enzymes (Figure 1c). Once synthesized, it is transported to the outer leaflet of the membrane by the ATP-binding cassette (ABC) transporter TarGH (also referred to as TagGH) [7], where it is linked to the peptidoglycan by LCP-type enzymes and further decorated with d-alanines likely transferred from the LTA polymer [8]. In addition, glycosyl modifications are added intracellularly (e.g. in *S. aureus;* Figure 1c) or, as more recently suggested, extracellularly (e.g. in *Listeria monocytogenes;* Figure 1d). Rhamnose polysaccharide biosynthesis proceeds similar to WTA synthesis and in *S. pyogenes* the required enzymes are encoded in the *gacABCDEFGHIJKL* gene cluster. Its synthesis is initiated by GacO, which, like TarO, transfers phospho-GlcNAc onto C_55_-P producing C_55_-PP-GlcNAc [9,10]. Next, the polymer is extended by a number of rhamnosyltransferases thought to include GacBCFG, transported to the outside of the membrane by an ABC transporter (GacDE) and linked to peptidoglycan by LCP-type enzymes (Figure 1e) [11,12^••^,^13^]. The rhamnose polysaccharide is also further modified on the outside of the cell with glycosyl groups (Figure 1e) [12^••^]. Lactococci also produce rhamnose-containing polysaccharides, that consist of two different components: a conserved rhamnan chain anchored to, and embedded within, the peptidoglycan layer and a more variable chain exposed at the bacterial surface, known as polysaccharide pellicle [11,14,15]. These two chains are thought to be produced separately and subsequently covalently linked together on the outside of the cell, forming a large heteropolysaccharide [16]. In contrast to WTA and rhamnose polysaccharide synthesis, a Wzx/Wzy-enzyme-dependent pathway has been proposed for the synthesis of the complex SCWP in *Bacillus anthracis*, which is based on bioinformatic predictions and analyses of mutant strains [17]. The *B. anthracis* SCWP is a polymer of β-ManNAc-β-GlcNAc-α-GlcNAc trisaccharide repeating units. In the proposed model, the trisaccharide repeating units are produced on a C_55_-P lipid carrier in the cytoplasm potentially using a TagO enzyme for the initial step, translocated across the membrane via a Wzx-type flippase, polymerized on the outside by a Wzy-type enzyme and attached to the peptidoglycan by LCP-type proteins (Figure 1f) [17, 18, 19, 20, 21,22^••^]. The SCWP backbone is further decorated with sugar residues, which are likely introduced before as well as after the transport of the trisaccharide subunits across the membrane (Figure 1f) [22^••^].

## General principles of multi-component transmembrane glycosylation systems

**Figure 2:**
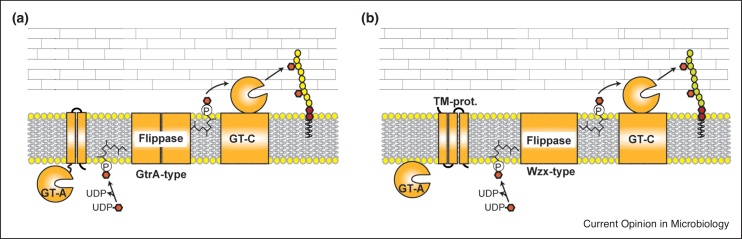
Compositions of multi-component transmembrane glycosylation systems. **(a)** Schematic representation of a multi-component transmembrane glycosylation system with a membrane-linked GT-A glycosyltransferase, a GtrA-type flippase (likely functioning as a dimer) and a GT-C fold membrane glycosyltransferase. Such systems are used for the LTA glycosylation process in *B. subtilis, S. aureus* and for both, LTA and WTA glycosylation in *L. monocytogenes,* as well as for the glycosylation of SCWPs such as for instance those produced by *L. lactis* and *B. anthracis*. **(b)** Schematic representation of a multi-component transmembrane glycosylation system with a cytoplasmic GT-A-fold glycosyltransferase, which binds to a separate membrane protein (TM-prot.) for efficient function, a Wzx-type flippase enzyme and a GT-C-fold membrane glycosyltransferase. Such a system has been proposed to be used for the extracellular glycosylation process of the rhamnose polysaccharide in *S. pyogenes.* Combinations of these two systems might also exist.

As mentioned above, TAs as well as complex SCWPs are often further modified with glycosyl groups. Type I LTA is synthesized on the outside of the cell; thus, glycosylation of the polymer must occur extracellularly. A model for the glycosylation of LTA has already been proposed in the 1980s (reviewed in Ref. [23]) but the enzymes involved have only been identified recently (Table 1). It has become apparent that similar extracellular glycosylation systems are also involved in the decoration of WTAs and complex SCWPs and are also important for the periplasmic modification of lipopolysaccharide and O-antigen residues in Gram-negative bacteria. They have been referred to as three-enzyme or three-component glycosylation systems [24^••^,25^••^,^26^], as the first characterized systems were composed of a membrane-linked GT-A-fold C_55_-P sugar-activating glycosyltransferase, which produces a C_55_-P-sugar intermediate, a flippase and a multimembrane-spanning GT-C- fold glycosyltransferase, which transfers the sugar from the lipid intermediate to the cell wall polymer (Figure 2a). However, recent studies have indicated that more than three proteins, as well as enzymes belonging to different protein families, can be involved in this process (Figure 2b), hence we propose to rename the extracellular glycosylation systems for TAs and complex SCWPs to multi-component transmembrane glycosylation systems. For instance, in place of a membrane-linked GT-A-type glycosyltransferase, a glycosyltransferase and a separate membrane protein can function together to produce the C_55_-P-sugar intermediate (Figure 2b). In many bacteria the flippase enzyme is thought to belong to the GtrA-type protein family but for some bacteria the involvement of Wzx-type family of flippases has been suggested (Figure 2b) [12^••^,^27^]. Finally, the multimembrane spanning GT-C-type glycosyltransferases, which transfer the sugar moiety from the lipid intermediate onto the cell wall polymer, can have low levels of similarity on the amino acid sequence level between different systems. They are integral membrane proteins and usually contain a conserved DxD or modified DxD motif within an extracellular loop [28]. The known GT-C-type glycosyltransferases involved in the modification of TAs and SCWPs are predicted to contain between 8–13 transmembrane helices (Table 2), which makes their identification using sequence similarity and membrane topology prediction tools difficult. However, using structure prediction programmes such as HHpred [29] the same structural homologs can be identified as top hits (analysis performed on September 7th 2020), namely the Arabinofuranosyltransferase AftD from *Mycobacteroides abscessus* subsp. *abscessus* (6W98_A) [30^••^], the 4-amino-4-deoxy-l-arabinose transferase from *Cupriavidus metallidurans* (5EZM_A) [31], the Dolichyl-diphosphooligosaccharide-protein glycosyltransferase subunit STT3A (6S7O_A) and STT3B (6S7T_A) from *Homo sapiens* and subunit 1 from *Saccharomyces cerevisiae* (6EZN_F) [32,33], the oligosaccharyltransferase from *Archaeoglobus fulgidus* (3WAJ_A) [34] and an oligosaccharyltransferase from *Campylobacter lari* (5OGL_A) [35]. These structural studies revealed important information on enzyme function and acceptor molecule binding and domains required for this. Some of the larger GT-C-fold glycosyltransferases, such as the *M. abscessus* AftD protein [30^••^] and potentially also the YfhO protein from *S. aureus* contain extracellular carbohydrate binding domains thought to be involved in acceptor cell wall polymer binding. Hence, we speculate that in some systems with smaller GT-C-fold glycosyltransferases the final transfer of the sugar to the cell wall polymer could potentially require multiple interacting proteins, with one protein essential for the sugar transfer (GT-C-type glycosyltransferase) and a second protein required for the recognition and binding of the acceptor cell wall polymer.

**Table 1:** Glycosyltransferases and predicted flippases required for glycosylation of LTA, WTA and complex cell wall polysaccharides

Organism	GT-A^a^	Putative flippase^a^	GT-C^a^	Sugar added	Acceptor	References
*L. monocytogenes* serotype 1/2a (10403S and EGD-e)	GtlA (Lmo0933)	GtcA (Lmo2549)	GtlB (Lmo0626)	Gal	LTA	[27,36^••^,^39^]
	CsbB (Lmo2550)	GtcA (Lmo2549)	YfhO (Lmo1079)	GlcNAc	WTA	[27,40,41,44]
*L. monocytogenes* serotype 4b (WSLC_1042)	GttA (AX24_02795)	Predicted GtcA (AX24_10700)	GtlB (AX24_00410)	Gal	LTA	[37^•^,^55^] and this review (prediction)
	GttA (AX24_02795)	Predicted GtcA (AX24_10700)	GttB (AX24_02800)	Gal	WTA	[37^•^,38^•^] and this review (prediction)
	GltA (AX24_11905)	Predicted GtcA (AX24_10700)	GltB (AX24_11900)	Glc	WTA	[43] and this review (prediction)
*L. monocytogenes* serotype 4c (F6214-1)	GtcB (GlcV; ACA53384.1)	GtcA (AMS35013.1)	GtcC (PmpA; ACA53385.1)	Gal	WTA	[45]
*B. subtilis* 168	CsbB (BSU08600)	GtcA (BSU38210)	YfhO (BSU08610)	GlcNAc	LTA	[36^••^]
	YkoT (BSU13390)	Unknown	YkoS (BSU13380)	Unknown	Unknown	[36^••^]
	YkcC (BSU12890)	Unknown	YkcB (BSU12880)	Unknown	Unknown	[36^••^]
*S. aureus* RN4220	CsbB (SAOUHSC_00713)	GtcA (SAOUHSC_02722)	YfhO (SAOUHSC_01213)	GlcNAc	LTA	[24^••^]
*L. lactis* NZ9000	CsdA (Llnz_00690)	CflA (Llnz_02975)	CsdB (Llnz_00695)	Glc	Rhamnan	[25^••^]
	CsdC (Llnz_03080)	CflA (Llnz_02975)	CsdD (Llnz_03075)	Glc	Polysaccharide pellicle	[25^••^]
	CsdE (Llnz_07820)	CflA (Llnz_02975)	CsdF (Llnz_07825)	Gal	LTA	[25^••^]
*B. anthracis* Sterne	GtsA (BAS5287)	GtsB (BAS5286)	GtsC (BAS5285)	Gal	SCWP	[22^••^]
*S. pyogenes* MGAS5005 (GAS M1-serotype)	GacI (M5005_Spy0610)	GacK^b^ (M5005_Spy0612)	GacL (M5005_Spy0613)	GlcNAc	Lancefield group A carbohydrate (GAC)	[9,12^••^]
*S. mutans* U159 (c-serotype)	RgpI (SMU.833)	Predicted (SMU.1546)	RgpH (SMU.832)	GlcNAc	Serotype c carbohydrate (SCC)	[56] and this review (prediction)

a GenBank Gene ID numbers are indicated in parentheses.

b GacK is a Wzx-type family flippase.

**Table 2:** GT-C-type glycosyltransferases and their predicted membrane topologies

Organism^a^	Enzyme	Domains^b^	Topology^c^	References
*L. monocytogenes* 10403S (1/2a)	YfhO (Lmo1079)	YfhO		[36^••^]
	GtlB (Lmo0626)	PMT-2		[36^••^]
*L. monocytogenes* EGD-e (1/2a)	YfhO (Lmo1079)	YfhO		[44]
*L. monocytogenes* WSLC_1042 (4b)	GttB (AX24_02800)	PMT-2		[38^•^]
	GltB (AX24_11900)	none		[43]
	GtlB (AX24_00410)	none		[37^•^]
*L. monocytogenes* F6214-1 (4c)	GtcC (PmpA)	PMT-2		[45]
*B. subtilis* 168	YfhO (BSU08610)	YfhO		[36^••^]
	YkoS (BSU13380)	none		[36^••^]
	YkcB (BSU12880)	PMT-2		[36^••^]
*S. aureus* RN4220	YfhO (SAOUHSC_01213)	YfhO		[24^••^]
*L. lactis* NZ9000	CsdB (Llnz_00695)	none		[25^••^]
	CsdD (Llnz_03075)	PMT-2		[25^••^]
	CsdF (Llnz_07825)	PMT-2		[25^••^]
*B. anthracis* Sterne	GtsC (BAS5285)	PMT-2		[22^••^]
*S. pyogenes* MGAS5005 (GAS M1-serotype)	GacL (M5005_Spy0613)	PMT-2		[12^••^]
*S. mutans* U159 (c-serotype)	RgpH (SMU.832)	Scs3p		[56]

a Serotype of strains are indicated in parentheses.

b PMT-2 = Dolichyl-phosphate-mannose-protein mannosyltransferase; Scs3p = Inositol phospholipid synthesis and fat-storage-inducing transmembrane protein.

c Topology model based on TMHMM server 2.0 analysis [57].

## Characterized multi-component transmembrane glycosylation systems

The proteins required for LTA glycosylation have now been identified in *Bacillus subtilis*, *S. aureus* and *L. monocytogenes* (Table 1) [24^••^,^27^,36^••^,37^•^,38^•^]. The GT-A-type glycosyltransferase GtlA of *L. monocytogenes* serotype 1/2a utilizes UDP-galactose to form a C_55_-P-galactose, which is transported across the membrane by the predicted GtrA-type flippase GtcA. GtrA-type proteins are small membrane proteins with 3–4 transmembrane helices and thought to function as dimers. Next, the galactose (Gal) residues are transferred onto the LTA backbone by the GT-C-fold glycosyltransferase GtlB [27,36^••^,^39^]. Interestingly, the GtlA homolog in the *L. monocytogenes* serotype 4b strain WSLC_1042, GttA, is required for modification of both WTA and LTA with Gal residues (Table 1) [37^•^]. In *B. subtilis* and *S. aureus,* CsbB, YfhO and GtcA have been identified as the GT-A-, GT-C-type glycosyltransferases and the GtrA-family flippase involved in the glycosylation of LTA with GlcNAc residues [24^••^,^27^,36^••^]. *B. subtilis* possesses two additional GT-A and GT-C pairs, YkcBC and YkoST, but their functions remain unknown [36^••^]. Surprisingly, the deletion of genes coding for CsbB (Lmo2550) and YfhO (Lmo1079) homologs in *L. monocytogenes* serotype 1/2a strains led to the absence of GlcNAc modifications on WTA and not LTA [36^••^,^40^,^41^]. This suggests that glycosylation of WTA takes place extracellularly in *L. monocytogenes* utilizing a multi-component transmembrane glycosylation system similar to the LTA glycosylation system [36^••^] (Figure 2a). The structure of WTA differs significantly between different *L. monocytogenes* serotypes (reviewed in Ref. [42]) and several other GT-A, GT-C glycosyltransferases and putative flippases have been shown to be required for the glycosylation of WTA in different *L. monocytogenes* serotypes (see Table 1) [27,36^••^,38^•^,^39^, ^40^, ^41^,^43^, ^44^, ^45^].

The glycosylation of more complex cell wall polysaccharides are also often accomplished by multi-component transmembrane glycosylation systems. For instance, in *Lactococcus lactis* genes *csdAB, csdCD* and *csdEF* encode GT-A and GT-C-type glycosyltransferase pairs, respectively [25^••^]. CsdEF are required for the galactosylation of LTA, whereas CsdAB and CsdCD are involved in the glucose modification of the rhamnan polymer and polysaccharide pellicle, respectively. Glycosylation of all three polymers depends on a single GtrA-type flippase enzyme, CflA, suggesting that different C_55_-P-sugar intermediates can be transported across the membrane by the same protein [25^••^]. While it has been suggested that the rhamnose polysaccharide of *S. mutans* is decorated with glucose residues intracellularly [46], based on structural predictions, one of the required proteins, RgpH, resembles a GT-C-fold membrane glycosyltransferase and a second protein, RgpI, is similar to membrane-bound GT-A glycosyltransferases such as CsbB. Hence, we hypothesize that in *S. mutans* the glucose residues on the rhamnose polysaccharide are also introduced extracellularly by a multi-component transmembrane glycosylation system and that the glycosyltransferases RgpI and RgpH potentially function together with the GtrA-type flippase protein SMU.1546 (Table 1, Table 2). In *S. pyogenes*, glycosylation of the rhamnan polymer has been proposed to occur on the extracellular side of the membrane [12^••^]. However, in this case, the GT-A-fold glycosyltransferase GacI and GacJ, a small membrane protein with three transmembrane helices, are thought to be involved in the efficient production of the C_55_-P-GlcNAc lipid intermediate (Figure 2b). Also, in contrast to the rhamnan polysaccharide glycosylation process in *L. lactis,* the lipid intermediate is thought to be transported across the membrane by the Wzx-type flippase GacK. Subsequently, GacL, a GT-C-fold glycosyltransferase, transfers GlcNAc to the rhamnose polysaccharide [9,12^••^]. A similar mechanism as described for *S. pyogenes* has been proposed for the biosynthesis and glycosylation of the enterococcal polysaccharide antigen (EPA) in *Enterococcus faecalis* [47]. The EPA backbone is a rhamnan hexasaccharide substituted with Glc and GlcNAc residues, to which TAs are covalently linked, forming the so-called EPA decorations. How the Glc and GlcNAc substitutions are introduced is currently unknown, but it has been proposed that these decorations are introduced extracellularly by multi-component transmembrane glycosylation systems.

For the synthesis of the *B. anthracis* SCWP, both intracellular and extracellular glycosylation systems are thought to be involved. It has been suggested that the glycosyltransferase GtsE uses UDP-Gal as substrate and transfers Gal onto the O4 position of the αGlcNAc in the lipid-bound C_55_-PP-αGlcNAc-βGlcNAc-βManNAc trisaccharide repeat within the cytoplasm of the cell (Figure 1f) [22^••^]. Precursor units are then transported to the outside of the membrane and a multi-component transmembrane glycosylation system composed of the GT-A-type glycosyltransferase GtsA, the GtrA-type flippase GtsB, and the GT-C-type glycosyltransferase GtsC, have been suggested to be responsible for the α-Gal modification on the O3 position of both GlcNAc residues [22^••^]. Interestingly, while the complete lack of Gal modifications and the lack of the β-Gal residues introduced by GtsE are tolerated, once the β-Gal has been added, the multi-component transmembrane system becomes essential [22^••^]. It has been suggested that this results from the toxic accumulation of the C_55_-PP-αGlcNAc-βGlcNAc-βManNAc intermediate substituted with β-Gal. The authors hypothesized that this intermediate cannot be polymerized leading to a depletion of the C_55_-P lipid carrier molecule, which is also required for other processes such as peptidoglycan synthesis [22^••^]. This makes the *B. anthracis* multi-component transmembrane glycosylation system one of the first essential systems described to date.

## Diverse cellular functions of glycosyl modifications on cell wall polymers

Many functions have been assigned to the glycosyl modifications on WTA, which include their importance for pathogenesis, immunorecognition, antibiotic resistance, attachment of cell wall hydrolases and serving as phage receptors and these have been reviewed elsewhere [48]. Complex SCWPs, which are usually linked to the peptidoglycan polymer, fulfil similar cellular functions as WTA. Consistent with this, glycosyl modifications on these polymers have also been reported to play a role in processes such as pathogenesis, resistance to cationic antimicrobial peptides, and to serve as phage receptors (reviewed in Refs. [49,50]). As mentioned above, one notable exception is the multi-component transmembrane glycosylation system in *B. anthracis,* which appears to be essential for the actual assembly of the complex SCWP and viability of the organism [22^••^]. A lot less is known about the cellular function of glycosyl modifications on LTA. The expression of the LTA glycosyltransferases CsbB and YfhO in *B. subtilis* is under the control of the alternative sigma factors σ^B^ and σ^X^ [51,52]. Expression of *csbB* is upregulated under environmental stress conditions such as salt, ethanol or oxidative stress in *B. subtilis* [53,54]. Expression of *S. aureus csbB* is also induced under salt stress in a σ^B^-dependent manner [24^••^]. The overexpression of the *L. lactis* glycosyltransferases CsdE and CsdF required for LTA glycosylation resulted in an increased resistance to nisin, while absence of these proteins led to decreased nisin resistance [25^••^]. Glycosyl modifications on LTA therefore seem to be required under stress conditions, however, further studies are necessary to understand the cellular function of LTA glycosylation in detail.

## Conclusions and important outstanding questions

In recent years it has become apparent that extracellular glycosylation systems are not only used for the decoration of LTA, but more widely used for the modification of different cell wall polymers. Such systems were initially referred to as three-component glycosylation systems, but we propose to rename them to multi-component transmembrane glycosylation systems to better reflect their diversity and that more than three proteins can be involved. One characteristic of such systems are GT-C-fold glycosyltransferases and the discovery of several different GT-C enzymes makes it now possible to bioinformatically predict such enzymes in other bacteria. Furthermore, the use of structure prediction programmes will aid in the identification of additional novel GT-C enzymes, which show only limited sequence homology to previously characterized enzymes. However, actual structural information of GT-C enzymes is needed to better understand their mechanism of action as well as sugar- and acceptor-molecule specificity. This could also clarify if some of the smaller GT-C enzymes require additional proteins for the recognition and binding of the acceptor cell wall polymer.

For complex SCWPs produced via a Wzx/Wzy-enzyme-dependent synthesis and polymerization process such as proposed for *B. anthracis,* it will be important to further investigate the mechanism behind the essentiality of the multi-component transmembrane glycosylation system and if this also holds true for other bacteria, which use similar SCWPs synthesis mechanisms. Similarly, for WTA in *L. monocytogenes* and rhamnose polysaccharide in other species, which are linked by LCP-type enzymes to the peptidoglycan layer, it will be interesting to determine at what point the extracellular glycosylation step takes place and how the activity of the GT-C enzymes is coordinated with the activity of LCP-type enzymes. Furthermore, for cell wall polymers, which are both glycosylated and d-alanylated, it remains to be determined if and how these two processes are coordinated.

While diverse functions have been ascribed to the glycosyl modifications on WTA, the cellular function of glycosyl modifications on LTA is less clear. Based on reports on the expression of genes coding for LTA glycosylation enzymes, such modifications might help bacteria survive under specific stress conditions such as osmotic stress, which warrants further investigation. It will be interesting to address if there are mechanisms to actively remove glycosyl residues from cell wall polymers once the stress subsides. Or alternatively, if it takes several generations until the sugar modifications are removed after gene expression is switched off. The salt-inducible LTA glycosylation process in *S. aureus* represents a good model to address such questions.

Finally, as exemplified in *B. subtilis,* the identification of the CsbB/YfhO system as LTA glycosylation system and the observation that additional gene clusters coding for GT-A and GT-C glycosyltransferase pairs are present in the genome, opens up interesting possibilities that other cell surface structures might be glycosylated using a similar system as used for the glycosylation of LTA. Alternatively, the LTA or potentially WTA or other minor cell wall polymers might be glycosylated with different sugars under specific growth conditions. It will be exciting to address such questions in future studies.

## Conflict of interest statement

Nothing declared.

## References and recommended reading

Papers of particular interest, published within the period of review, have been highlighted as:

• of special interest
•• of outstanding interest
